# Evaluation of the accuracy of new modalities in the assessment and classification of lumbar lordosis: A comparison to Cobb's angle measurement

**DOI:** 10.1016/j.heliyon.2023.e18952

**Published:** 2023-08-06

**Authors:** Hassane Kheir Eddine, Sahera Saleh, Joseph Hajjar, Hayat Harati, Zeina Nasser, Alban Desoutter, Elie Al Ahmar, Elias Estephan

**Affiliations:** a Neuroscience Research Center, Faculty of Medical Sciences, Lebanese University, Beirut, Lebanon; b LBN, University Montpellier, Montpellier, France; c School of Engineering, Holy Spirit University of Kaslik, Jounieh, Lebanon; d Faculty of Arts and Sciences, Holy Spirit University of Kaslik, Jounieh, Lebanon

**Keywords:** Lumbar lordosis, Diagnostic accuracy, Cobb's angle, Lateral radiograph, New methods

## Abstract

**Background:**

Because of the association of lumbar lordosis with some clinical conditions such as low back pain, the chiropractic field has emphasized the significance of evaluating the lumbar lordotic status, by measuring Cobb's angle, regarded as the radiological gold standard, for the assessment of lumbar lordosis, on lateral radiographs. However, research has shown that this technique has some considerable drawbacks, mostly in terms of low accuracy and high variability between clinicians when compared with other radiological modalities. The main objective was to compare the diagnostic accuracy of newly established radiological measurements with one of Cobb's angle methods, for the characterization of lumbar lordosis status in a sample of Lebanese patients aged 15 and above.

**Material and methods:**

This retrospective single-center study consisted of measuring Cobb's L1-S1 and Cobb's L1-L5 angles, along with the novel established measurements which are the derivative and the normalized surface area, on 134 lateral radiographs of the lumbar spine of Lebanese patients aged fifteen years old and above, gotten from the Radiology department at Zahra'a’s Hospital in Beirut, performed by two observers using MATLAB. Inter-rater agreement was assessed by calculating the Intra-class correlation coefficients. Spearman correlation was analyzed between both Cobb's angle methods and with the derivative and normalized area respectively. 54 patients of the sample were diagnosed by two radiologists, according to their LL status. ROC curve analysis was performed to compare the diagnostic accuracy of the four techniques used. Data were analyzed with IBM SPSS Statistics 23.0 (NY, USA); *P* < 0.05 was considered statistically significant.

**Results:**

According to the ROC curve analysis the new methods, which are the derivative and the normalized surface area, displayed lower diagnostic accuracy (AUC_derivative_ = 0.818 and 0.677, AUC_surface area_ = 0.796 and 0.828) than Cobb's L1-L5 (AUC_L1-L5_ = 0.924 and 0.929 values) and L1-S1 (AUC_L1-S1_ = 0.971 and 0.955) angles, in the characterization of hypo and hyperlordotic patients, respectively, in our Lebanese sample consisting of patients aged 15 and above, because of their lower area under the curve's values compared to the traditional Cobb's techniques. The Cobb's L1-S1 has shown to have the highest diagnostic accuracy among the four methods to characterize normal patients from hypo and hyperlordotic ones, by referring to its highest area under the curve's values. However, the sensitivity of Cobb's L1-L5 angle in characterizing hyperlordotic patients was similar to the one of the normalized surface area with a value of 100%.

Conclusion: among the four modalities, the new methods didn't show a better diagnostic accuracy compared to the traditional modalities. Cobb's L1-S1 displayed the highest diagnostic accuracy despite its drawbacks. Further prospective studies are needed to validate the cut-offs obtained for Cobb's L1-S1 angle in our sample.

## 1 Introduction

Lumbar Lordosis (LL) constitutes the ventral curvature of the lumbar spine, along the wedging between the lumbar vertebral bodies and the intervertebral disks [[1], [2], [3]]. This anatomical parameter participates intimately in the settlement of a balanced spinal profile, in addition to thoracic kyphosis and sacral inclination [4]. Most researchers, indeed, found that greater lordosis usually relates to more pronounced thoracic kyphosis, although other controversial cases were reported in which there was reduced thoracic kyphosis. Small lordosis angles are usually correlated with reduced thoracic kyphosis; however, cases of reduced lumbar lordosis with increased thoracic kyphosis have also been displayed [5,6]. In terms of general facts regarding LL, it seems to start forming in early childhood, increases during adolescence [7], and hit its full development in the period of spinal maturation after the age of 15 [8], with a higher probability of LL abnormality appearing in female participants, compared to male individuals [6,9].

The measurement of the lumbar lordotic angle (LLC) has been an important concern in the chiropractic field; because it exerts variations on the clinical setting of the patient. It has been demonstrated to be a strong mechano-morphological predictor of some lumbar pathologies [10], and a potential determinant in lower back pain (LBP) [11].

LBP is considered a disease with a high incidence rate, where more than 60% of individuals experience it at least once during their lifetime. Additionally, it is considered to be one of the top leading causes of professional and social disability worldwide [12]. Laird et al. stated, in a systematic review conducted in 2014, that people with LBP have reduced lumbar range of motion and proprioception, and move more slowly in comparison to individuals who do not have LBP, regardless of the onset time of the lumbar lordotic pathology, whether prior or after LBP [11]. Also, a prior study conducted by Kim et al., in 2006, showed that patients at high risk of developing lower back pain present with limited lumbar lordosis [13]. Considering the burden exerted by this condition, it is important, therefore, to assess the LLC.

Besides chiropractors, experts in other branches of rehabilitation sciences, specifically Orthopedics, Orthotics, and Physiotherapy, are deeply concerned about assessing the LLC as well [2]. Indeed, when treating adolescents with Scheuermann's kyphosis or idiopathic scoliosis, it is crucial to evaluate the changes in some spinopelvic parameters, including lumbar lordosis. This evaluation is essential in determining the planning of the treatment, as well as its effectiveness in the long run [[14], [15], [16]].

The quantitative assessment of the lumbar lordotic curvature involves the radiographic evaluation of some angular measurements in different ways [17,18] or other physical parameters to quantify the LLC, based on a sagittal image of the lumbar spine [2]. Besides radiographic measurements, non-radiographic modalities are used to assess quantitatively the LLC, such as the spinal mouse method which could be an alternative when radiographs are not feasible, but it consists of measuring soft tissue contours rather than the lordosis itself [19]. Despite exposure to radiation which represents an issue to a certain extent in radiology, Cobb's angle, also known as the lordotic angle [20], is widely regarded as the gold standard [17,21,22]. However, the authors have shed light upon some relevant limitations for this angle. Indeed, it has a poor accuracy in identifying local curvature changes [2,9,22]; whereas, for the same angle value, there may be different lordotic curvature magnitudes [2]. Besides, some papers stated inter-rater variability in terms of mean and standard deviation [17,20,23,24].

Because of these limitations and to overcome them, two new methods that offer a different way of evaluation of the lumbar lordotic curvature are proposed herein. These methods consist of quantifying the surface area delineated by the lordotic lumbar curvature and computing the average derivative of the curve fitted to the extension along the fourth and fifth lumbar vertebrae and the first sacral one. The measurements were performed by running out an established code on MATLAB [25], to assess Lumbar Lordosis on two-dimensional radiographs; measurements would be digitalized and more standardized and results would be more accurate and reliable, unlike the usual tracing and measurement done, manually, and displaying higher risks of errors [23].

The main objective of our study was to evaluate the diagnostic accuracy of the proposed methods in comparison to that of the digitalized gold standard, towards the diagnosis of LL in Lebanese patients aged 15 years old and above. We aimed to assess their correlation with Cobb's L1-S1 and L1-L5 angles. Another objective of this study was to compare the two applied versions of Cobb's angle technique in diagnosing LL, in terms of reliability and diagnostic accuracy as well.

## 2 Material and methods

### 2.1 Population and sample size

This retrospective single-center study included a total number of 182 standing lateral lumbar spine radiographs of Lebanese patients admitted to Zahraa Hospital in Beirut, Lebanon after attaining the Institutional review board (IRB) approval from the IRB committee of the hospital (reference number: 2/2023). Radiographs have been collected conveniently, after having obtained patients' consent to participate in the study and to have their radiographs published in our manuscript. The database size, available for the study, was either comparable or higher to that found in clinical evaluations in the same domain [18,19,26,27], thus, it was considered acceptable. Lateral radiographs were assessed for eligibility. Effectively, images of patients aged 15 and above, were enrolled, to make sure that they have attained their full lumbar lordosis development [8]; images must have been of good quality and display no associated vertebral pathology as well, to be included. Otherwise, exclusion criteria were the following: (a) patients aged less than 15, (b) missing documentation about the gender and age of the patient, (c) radiographs of bad quality, and (d) radiographs displaying vertebral pathologies such as spondylolisthesis. The assessment of patients’ radiographs was done by a specialist according to the eligibility criteria, leaving us with a total number of 134 participants. The retrospective approach was used in this study to avoid the ethical issue of patient irradiation.

### 2.2 Data collection

Each eligible radiograph was subjected to four measurements, which were: Cobb's (L1-L5) angle, Cobb's (L1-S1) angle, the derivative, and the surface area, using MATLAB software. A code was established using MATLAB (an acronym for MATrix LABoratory), a high-performance and powerful tool for technical computing, to make all the measurements at once on each radiograph [25].

#### 2.2.1 Cobb's angle

**Fig. 1:**
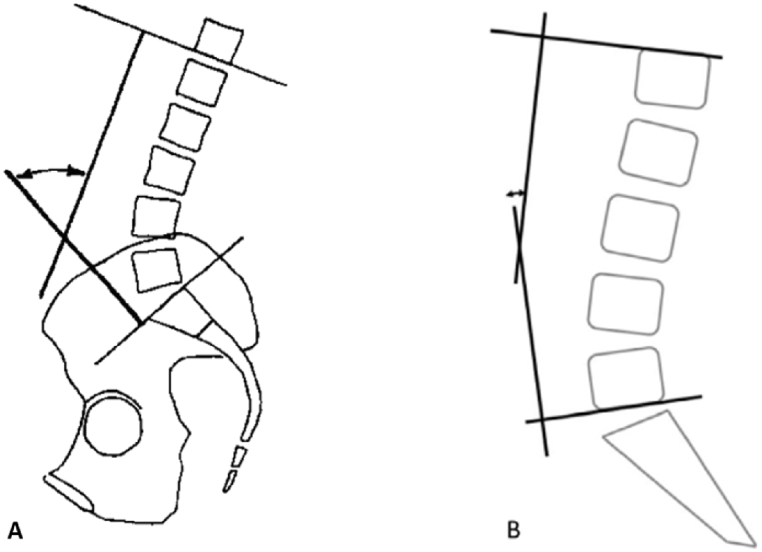
Radiographic measurement of Cobb's angle, demarcated by the lines located at the upper endplates of L1 and S1 (A) or upper endplate of L1 and lower endplate of L5 (B) [28].

In the most common version of its analysis, a line was demarcated through the superior endplate of the first lumbar vertebra (L1), and a second one is drawn parallel to the superior endplate of the sacral base (S1) or the inferior endplate of the fifth lumbar vertebra (L5). Then, perpendiculars are traced, and the angle at the intersection is precisely measured [2,28]. Fig. 1 displays accurately Cobb's angle technique, measured conventionally. Although the L1-S1 definition of LL is recommended rather than others, because of the important contribution of the L5-S1 disk in LL [2,29], we decided in the study to measure Cobb's L1- S1 angle (Fig. 1A) and Cobb's L1- L5 angle (Fig. 1B), because the latter has shown to be more reliable [24,30].

#### 2.2.2 Surface area and derivative

The surface area of the lordotic curve is the area bounded by the lumbar lordosis, which means the line that is traced along the posterior side of the vertebral column starting from L1 to S1.

The derivative is the average slope englobing all demarcated points along the dorsal lining of the fourth and fifth lumbar vertebrae (L4 and L5, respectively) and S1.

**Fig. 2:**
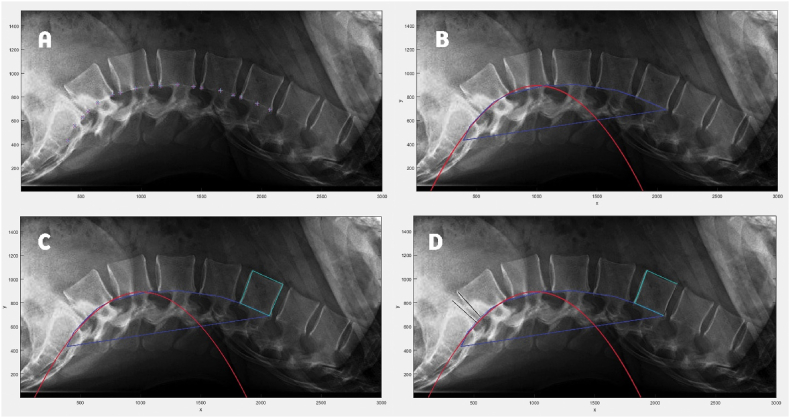
The different steps displayed successively from A to B while running out the code on MATLAB to get the required measurements; whereas A displays the fixed dots that form the lumbar lordotic curve from L1 to S1; B shows the binomial fitted curve in red and the demarcated surface that has been calculated then normalized to the surface of L1, as shown in C. finally, D displays the lines (in black) that pass through the upper endplates of L1 and S1 and the lower endplate of L5.

The measurement of these aforementioned parameters was performed as described in the following: 18 points were fixed along the curvature that extends from S1 to L1, in a way that 3 points were plotted on each vertebra, on the caudal side, midpoint, and rostral side (Fig. 2A). Then, the nine points lining the posterior vertebrae L4, L5, and S1, were fitted into a polynomial curve of the second degree, marked in red (Fig. 2B), by using the fit function. The derivative was therefore calculated by using differentiation, and its value at each of the nine plotted points was determined. The average of the derivatives at each of the individual points was finally reported. The surface delineated by the blue line is normalized to the surface of L1, which was demarcated by the light blue square joining the 4 tips of the vertebra (Fig. 2C) to eliminate any error caused by differences in magnification and distortion among the images. The measurements of the derivative and the normalized surface area were then established.

Following that, the lines passing through the superior endplate of L1, the superior endplate of S1, and the inferior endplate of L5 (Fig. 2D), were traced after plotting points on the upper and lower extremes of each S1 and L5 appropriate endplates. Then, measurements of Cobb's angle considering L1-L5 and L1-S1 were respectively obtained.

Two observers made their measurements on the original dataset of images, in the same way. Among the 134 patients, 54 have been referred to two radiologists and have been attributed the same diagnosis by both in terms of lumbar lordosis status.

### 2.3 Statistical analysis

Data were analyzed with the IBM Statistical Package for the Social Sciences (SPSS) (version 23.0) (NY, USA); *P* < 0.05 was considered statistically significant. The descriptive analysis was realized in terms of age and gender. The Inter Class Correlation (ICC) coefficient was calculated to test the inter-examiner reliability of each of the four measurements performed by two observers. Following this, Spearman correlation was assessed between the values obtained from each of Cobb's L1-L5 angle and Cobb's L1-S1 angle respectively, and those of the derivative and normalized area, across the whole sample. Among the diagnosed patients, the Kruskal-Wallis test was run out to analyze the four measurements in terms of the lumbar lordosis status. Two Received Operating Characteristic (ROC) curves for each of them were drawn: between hypo-lordosis and normal lordosis and between normal lordosis and hyper-lordosis. The area under the curve (AUC) values, the main indicator of the level of diagnostic accuracy, were obtained for the new modalities and Cobb's techniques and compared to each other. The specificity (SP), sensitivity (SE) calculation, and respective cut-off points were determined according to the Youden Index.

## 3 Results

After the eligibility assessment, 134 patients' radiographs were subjected to the four measurements described previously, performed by two observers. 54.5% of the patients were female and the mean age valued 49.28 years (SD = 17.28, range = 15–88). Inter-examiner calibration of all measurements showed excellent agreement between observers, with ICCs that exceed 0.90 [31] in 95% confidence intervals, according to Table 1; while the highest ICC was that of the derivative (ICC_Derivative_ = 0.993 in 95% (0.984–0.996)).

**Table 1:** Intra Class Coefficients to assess inter-rater agreement, in terms of Cobb's L1-L5 angle, Cobb's L1-S1 angle, derivative and normalized area.

Measurements	ICC	CI 95%	
		Upper bound	Lower bound
Cobb's L1-L5 angle	0.988	0.983	0.992
Cobb's L1-S1 angle	0.980	0.925	0.992
Derivative	0.993	0.984	0.996
Normalized Area	0.976	0.966	0.983

ICC: Intra-class coefficient; CI: Confidence interval.

The new modalities measurements were revealed to be significantly correlated with Cobb's values, as displayed in Table 2; there was a positive moderate correlation with the derivative and the normalized area (Spearman r = 0.67, p-value <0.0001; r = 0.69, p-value <0.0001, respectively). This correlation was a bit weaker and regarded as fair to moderate between Cobb's L1-S1 and the derivative (Spearman r = 0.598, p-value <0.0001); whereas, it was stronger between Cobb's L1-S1 measurement and the normalized surface area, and it was considered moderate to strong (Spearman r = 0.737, p-value <0.0001) [32].

**Table 2:** Spearman correlation analysis of Cobb's angle with the derivative and normalized area.

N = 134	Cobb's (L1-L5) angle		Cobb's (L1-S1) angle	
	r	*P-value*	r	*P-value*
**Derivative**	0.670	*<0.0001*	0.598	*<0.0001*
**Normalized area**	0.690	*<0.0001*	0.737	*<0.0001*

N: Total number of participants.

r: Coefficient correlation.

*P-value* <0.05 is significant.

A group of 54 patients was admitted to two radiologists who diagnosed them in terms of lumbar lordosis status; there were 25 hypolordotic patients, 18 patients with normal lordosis, and 11 hyperlordotic ones. Table 3 displays the medians of the four measurements performed on each radiograph, showing significant differences among the categories of LL, after having run out the Kruskal-Wallis test. We notice a common increase in values of each modality from the hypo-lordosis status to the hyper-lordosis status.

**Table 3:** The display of the Cobb's angle, derivative and normalized area across lumbar lordosis categories in a sample of 54 patients, diagnosed by two radiologists.

	Lumbar Lordosis N’ = 54			*P-value*
	Hypolordosis (n = 25)	Normal lordosis (n = 18)	Hyperlordosis (n = 11)	
**Cobb's angle L1-L5**^†^	23 [10]	37 [10]	64 [13]	*<0.0001*
**Cobb's angle L1-S1**^†^	35 [11]	48 [13]	55 [11]	*<0.0001*
**Derivative**^†^	0.16 (0.17)	0.28 (0.17)	0.43 (0.25)	*<0.0001*
**Normalized area**^†^	2.57 (0.72)	3.37 (1.24)	4.26 (1.54)	*<0.0001*

*P-value <0.05 is significant*.

† Median (Interquartile).

Then we performed the ROC curve analysis, to assess the diagnostic accuracy for each method in terms of hypo and hyperlodotic status, depending upon their respective AUCs (Table 4). It seemed that the new methods have had acceptable to excellent levels of diagnostic accuracy [33] for the characterization of hypolordosis (AUC_Derivative_ = 0.818 and AUC_Area_ = 0.796), but a variable level of diagnostic accuracy towards the detection of hyperlordotic cases. According to the AUC value, the derivative's accuracy was regarded as less than acceptable (0.677) and was proven to be less than that of the normalized area (0.828).

**Table 4:** Display of the area under the curve, as well as the sensitivity, specificity, and respective cut-off for each diagnostic modality used in the study, obtained by the Youden index calculation, to show the ability of each one in discriminating between hypolordotic and normal individuals on one hand, and between normal and hyperlordotic individuals on the other hand.

	Cobbs L1S1	Cobbs L1L5	Derivative	Area
**Hypo-lordosis/Normal Lordosis**				
**AUC**	**0.971**	**0.924**	**0.818**	**0.796**
**SE**	**100%**	**84%**	**84%**	**80%**
**SP**	**83.3%**	**88.9%**	**66.7%**	**72.2%**
**Cut-off**	**42°**	**30°**	**0.24**	**2.94**
**Normal Lordosis/Hyper-lordosis**				
**AUC**	**0.955**	**0.929**	**0.677**	**0.828**
**SE**	**90.9%**	**100%**	**54.5%**	**100%**
**SP**	**94.4%**	**83.3%**	**83.3%**	**66.7%**
**Cut-off**	**59°**	**41°**	**0.41**	**3.59**

AUC: Area under the ROC curve; SE: sensitivity, SP: specificity.

Despite the obtained results presented by the other methods, Cobb's L1-S1 angle still displayed the highest diagnostic accuracy in terms of its AUC and was admitted to be outstanding [33] in the diagnosis of hyper and hypolordosis (0.971 and 0.955 respectively). Cobb's L1-L5 angle showed better accuracy than the new methods as well, with outstanding AUC values for the diagnosis of hypo and hyperlordosis (0.924 and 0.929, respectively).

For the diagnosis of hypolordosis, Cobb's angle L1-S1 showed the highest sensitivity (100%), while Cobb's L1-L5 showed the highest specificity (88.9%); both were still more sensitive and specific than the derivative (SE = 84%, SP = 66.7%) and the normalized area (SE = 80%, SP = 72.2%). As for the diagnosis of hyperlordosis, the highest sensitivity was expressed by Cobb's L1-L5 angle and the normalized area (SE = 100%), while the derivative was the least sensitive (SE = 54%). The highest specificity was attributed to Cobb's L1-S1 angle (94.4%), while the least specific method was the area (SP = 66.7%). The Youden index calculation could provide us with the appropriate cutoff for each category, in a way that we obtained normal range values for each modality. Hence, according to our analysis, normal lordotic patients in the sample would have a Cobb's L1-S1 angle between 42° and 59°, a Cobb's L1-L5 angle between 30° and 41°, a derivative between 0.24 and 0.41, and a normalized area between 2.94 and 3.59.

## 4 Discussion

Cobb's angle technique is still regarded as the radiologic gold standard tool for the assessment of lumbar lordosis despite the emergence of many studies that attempted to introduce alternative methods and compare them to Cobb's technique. These techniques were similarly based on manual tracings, made upon two-dimensional (2D) radiographs, and proved to be more reliable than Cobb's angle technique, among different observers [18,24,25]. The lack of reliability attributed to Cobb's method is mainly related to the unevenness of the vertebral endplates' architecture which is not always regular and may alter the exact tracing of the lines when done manually [2]. In this study, we measured Cobb's L1-L5 angle along with Cobb's L1-S1 because it was proven in the literature to be more reliable than the latter [24,27,30].

To overcome the limitation attributed to Cobb's technique, concerning the restriction of the measurement upon 2 vertebras out of 5 or 6 [2], we decided to establish two novel methods, different from those described in the literature now: the surface area and the derivative. The surface area delineated by the posterior contour of the lumbar vertebras and S1 (Fig. 2B), takes into account all lumbar vertebras, unlike Cobb's technique. Hence, we hypothesized that it would be a better way to assess the LL, by following the LLC and approximatively its real direction in a 2D plan.

On the other hand, the derivative was calculated based on the most demarcated region of the lumbar lordotic curve, which is the most relevant indicator of the lumbar lordotic status of the patient, when assessed on a 2D lateral radiograph [8]. These methods were established to get a better definition of the LLC; and the use of MATLAB was intended to provide a computerized way to assess spinal parameters, unlike the usual manual tracing and measurement of angles, by using protractors, as displayed in the previous papers [18,19], to facilitate the measurements, avoid potential manual errors that could come out of it, and time waste [23].

On behalf of the positive significant correlation obtained and exhibited by the new methods to Cobb's L1-L5 and L1-S1 angles (Table 2), Chen, in 1999, found a similar correlation between Cobb's L1-L5 angle and the vertebral centroid lumbar measurement [27]; whereas, Okapala et al. (2018) revealed a better correlation of the Cobb's L1-L5 to the Harrison Posterior Tangent Method (r = 0.936) as compared to the surface area and derivative [19]. According to Chen in 1999 [27], Cobb's L1-S1 displayed a significant correlation to the vertebral centroid measurement. This correlation was also similar between Cobb's L1-S1 and the normalized area.

The main purpose of the study was to find a technique with the highest diagnostic accuracy toward LL status. For this reason, 54 patients have been diagnosed by two radiologists, each and then we performed the ROC curve analysis involving each diagnostic tool used in terms of LL status. Cobb's L1-S1 angle technique had the highest diagnostic accuracy in the depiction of hypo and hyper-lordotic patients, as compared to the new methods and Cobb's L1-L5 angle. This could be explained by the fact that the diagnosis given by radiologists was based on the gold standard, which we have performed as well in a more standardized way by using MATLAB. In consequence, this led to a high conformity between our measurements and the diagnosis given by the radiologists; not to mention, the high reliability exhibited by Cobb's measurements between the two observers.

The low accuracy of the derivative, revealed by its low sensitivity expressed towards the diagnosis of hyperlordosis (54%), is possibly related to the fact that this technique relies upon the lower lumbar spine compartment, which means at the level of L5 and S1; unlike Cobb's technique and the surface area which take into account a bigger portion of the lumbar spine involved in lumbar lordosis. Therefore, fewer errors would occur. This may be the reason why the normalized area displayed a high sensitivity in diagnosing hyperlordosis (100%) in our study sample. We propose, to have better precision in the characterization of the true lumbar lordotic curvature, to perform the new measurements on three-dimensional images instead of 2D pictures, which would provide better demarcation of the vertebra and a more credible orientation of the lumbar lordotic curvature, to get more precise values and fewer errors. Till then, Cobb's L1-S1 would be the best modality to apply on lateral radiographs to diagnose LL status.

Moreover, there was the application of both versions of Cobb's technique which consisted of measuring the L1-S1 and L1-L5 angles. Cobb's L1-S1 angle proved to be as reliable as the L1-L5 angle (ICC_L1-S1_ = 0.988 and ICC_L1-L5_ = 0.98), unlike previous studies, where the L1-L5 angle displayed higher reliability than L1-S1 angle [23,27,30], and where L1-S1 angle's ICC was inferior to the one obtained in this research [24]. This could be explained by the use of accurate software that helped to overcome the errors produced by the manual tracings and measurements and provided a more standardized manner to perform even the gold standard method in its two commonly used versions. The aforementioned idea could be regarded as a strong point in the study. And there are other points of strength. One of them resides in the fact that no previous one has aimed to assess and compare the measurement validity of methods previously described in the literature for diagnosing lumbar lordosis on lateral radiographs, which adds novelty to this research. Another one was displayed in the diagnosis in terms of LL status that was made by two radiologists instead of one, to make it more credible.

As for the limitations of this study, they reside in many facts. Indeed, the sample size was relatively small, especially in terms of the number of patients having been diagnosed in terms of their lumbar lordotic status, by the radiologists (54 patients); and the numbers of patients in the three categories of LL status were not equivalent. It was a retrospective and single-center study and the cases were conveniently gathered. All these factors weaken our results in terms of accuracy and generalizability. Hence, further prospective studies need to be elaborated on larger sample sizes, with an increased number of diagnosed patients, to make our results more generalizable, more valid and even to validate the cut-offs obtained in terms of Cobb's L1-L5 and Cobb's L1-S1 angles, obtained in our sample.

## 5 Conclusion

Despite the new methods' significant correlation to Cobb's method, which is the gold standard for the assessment of Lumbar lordosis, and their high inter-rater reliability, we concluded that the newly established measurements in our study, which are the surface area and the derivative, displayed a diagnostic accuracy lower than the one of Cobb's angle methods. Cobb's L1-S1 showed to have the highest diagnostic accuracy in the characterization of LL, as compared to Cobb's L1-L5 angle and to the new methods, because of the highest values of AUC in the characterization of hypo and hyper-lordotic patients. However, the surface area measurement has had the highest sensitivity in depicting hyperlordotic patients, similar to Cobb's L1-L5 angle. Moreover, Cobb's L1-S1 proved to be as reliable as Cobb's L1-L5 angle, unlike findings in previous studies. This study could initiate the use of MATLAB as a better way to measure Cobb's technique and eventually other parameters, for a simpler and more accurate way of assessment, unlike manual tracings and measurements. However, future prospective studies are needed to be performed on a larger sample, to obtain more accurate and generalizable results and to validate clinically the cut-offs of Cobb's technique obtained from the ROC curve analysis in this study.

## Author contribution statement

Hassane Kheir Eddine; Sahera Saleh: Performed the experiments; Analyzed and interpreted the data; Wrote the paper. Joseph Hajjar: Performed the experiments; Analyzed and interpreted the data; Contributed reagents, materials, analysis tools or data; Wrote the paper. Hayat Harati; Zeina Nasser; Alban Desoutter: Analyzed and interpreted the data; Contributed reagents, materials, analysis tools or data. Elie Al Ahmar; Elias Estephan: Conceived and designed the experiments; Performed the experiments; Analyzed and interpreted the data; Contributed reagents, materials, analysis tools or data; Wrote the paper.

## Data availability statement

Data associated with this study has been deposited at 10.6084/m9.figshare.21511575.

## Additional information

No additional information is available for this paper.

## Declaration of competing interest

The authors declare that they have no known competing financial interests or personal relationships that could have appeared to influence the work reported in this paper.
